# S.W. Vascular Surgeons

**Published:** 1991-06

**Authors:** 


### South West Vascular Surgeons

### Meeting of the South West Vascular Surgeons at Morriston Hospital, Swansea on 23 February 1990.

### THE RADIOLOGY AND BIOCHEMISTRY OF ACUTE ISCHAEMIA: AIDS TO DIAGNOSIS

**D.R. Bird**
**Withybush General Hospital, Haverfordwest.**

The mortality from acute intestinal ischaemia is extremely high and the only hope for the patient is an early laparotomy with thrombo-embolectomy or vascular reconstruction, together with bowel resection.

Delay can lead to irreversible bowel infarction, but is often caused by uncertainty about the diagnosis since the early symptoms are vague and there are few specific laboratory tests.

Two recent cases will be presented together with a review of 60 cases in Withybush General Hospital and the combined Edinburgh Hospitals. The value of the easily available serum amylase level is under estimated and in combination with the white cell count and serum bicarbonate level forms a most helpful diagnostic triad.

There are also recognisable but often unrecognised diagnostic changes on a supine abdominal.

The clinical history and examination, taken with the radiological changes and the diagnostic triad of biochemical tests can frequently lead to an earlier diagnosis, and underline the need for early laparotomy.

### THE ROLE OF INTRAVENOUS STREPOKINASE IN ACUTE LIMB ISCHAEMIA

**J.J. Earnshaw, C. Cosgrove, D.C. Wilkins and B.P. Bliss. Plymouth**

Intravenous streptokinase (IVSK) infusions (100,000 u/h) have been given to 48 patients with 50 episodes of acute limb-threatening arterial ischaemia. The patients were selected as unlikely to benefit from surgery and included 17 thromboses, 14 emboli and 19 graft occlusions.

Complete lysis was achieved in 17 (34%) patients, and was better for emboli (50%) and graft occlusions (47%) than acute thromboses (6%). Final outcome after 30 days was limb salvage 60%, amputation 24% and death 16%, but this was achieved only after nine patients without lysis had vascular reconstructive surgery. One patient died from a stroke.

In conclusion IVSK has a role in acute limb ischaemia if surgery is inappropriate and intra-arterial thrombolysis unavailable. In particular, selected patients with emboli or graft occlusions without a neurological deficit may be most suitable.

### ARTERIAL EMBOLECTOMY: A LONG TERM PERSPECTIVE

**K. Varty, J.A. St. Johnston, G. Beets, W.B. Campbell. Royal Devon and Exeter Hospital, Exeter**

This study reviewed 113 elderly patients after embolectomy over a 10 year period, (median age 77, M:F 46:47). The 30-day mortality was 43%. This was associated with, age >80yrs., failed embolectomy, and aorto-iliac occlusions ($P<0.05$).

Median long term survival was 29 months, (range 1–126), with most deaths in the first year, and a good survival after this. The most common subsequent vascular event in these patients was stroke (10) with very few further limb emboli.

There was no difference in late mortality and vascular events between 28 patients on long term warfarin and 26 patients not anticoagulated.

Embolectomy has a high mortality in elderly patients, but there are patients with good long term survival. The value of anti-coagulation for these patients is unresolved, and requires further study.

### EXPERIENCE WITH THORACO-ABDOMINAL ANEURYSMS

**M. Horrocks**
**Bristol Royal Infirmary**

Between 1984 and 1989 28 aneurysms were repaired using a thoraco-abdominal approach for disease at or above the renals. Diagnosis was confirmed by C.T. scan and arteriography. Visceral and renal artery vessels were re-implanted by the Crawford technique or by use of a long anterior tongue of aorta and an oblique anastomosis.

Aneurysms starting at the left subclavian had the highest morbidity and mortality, those starting at or below the diaphragm having few complications. Renal function was protected by Inosine and renal failure was not a major problem. Spinal perfusion was protected where possible, only one patient developing a paraplegia. There were 4 early deaths and 3 patients have subsequently died. 5 year survival is approximately 60%.

### THE PRE-OPERATIVE DIAGNOSIS OF INFLAMMATORY ANEURYSMS

**W.G. Tennant, M. Horrocks**
**Vascular Studies Unit, Bristol Royal Infirmary**

Inflammatory aneurysms (I.A.), can be difficult and hazardous to repair, and have a high operative mortality. Accurate pre-operative diagnosis is essential to plan operative repair.

Serological techniques, such as plasma viscosity, have been used to make the diagnosis of inflammatory change. This, in common with other markers currently under investigation lacks specificity, and gives no indication of the anatomical extent of the fibrosis.

Ultrasound and computerised tomography are also of limited use, and diagnostic features of are present in fewer than half the cases subsequently confirmed as inflammatory aneurysms.

An ongoing prospective study in the use of magnetic resonance imaging as a highly accurate indicator of the presence and extent of inflammatory aortic aneurysm was described.

### CHYLOUS ASCITES FOLLOWING AORTIC ANEURYSM SURGERY

**C.A.C. Clyne, C.C. Wilmshurst, R. Sanger**
**Torbay Hospital, Torquay**

Chylous ascites is a rare entity and post-operative chylous ascites appearing after surgery for aortic aneurysms has only been recorded in the literature thirteen times in the past.

We present a case of a man who underwent a difficult, elective, ressection of an aortic aneurysm and proceeded to develop chylous ascites, which was treated ultimately with a Leveen Peritoneo-Venous Shunt. Ultimately the chylous ascites was controlled but the patient died of septicaemia, probably relating to the shunt itself.

### AORTIC RUPTURE IN PATIENTS WITH AN AORTIC GRAFT

**Jack Collin, Peter Lamont, Peter J. Morris**
**John Radcliffe Hospital, Oxford**

Aortic rupture is an uncommon late complication of abdominal aortic aneurysm repair. We present three patients who had undergone replacement of abdominal aortic aneurysms many years previously who presented with abdominal aortic rupture. One patient had developed an aneurysm between the renal arteries and the proximal end of the graft and it was this area of the native aortic wall which had ruptured. The other two cases arose through the development of false aneurysms at the site of Dacron to Dacon anastomoses where a section of the graft had been excised at the time of original surgery, presumably because the graft had been found to be too long immediately after insertion. One patient had an aorto-duodenal fistula and the other ruptured his false aneurysm retroperitoneally. We conclude that in aortic surgery there is no statute of limitation and the sins of a surgeon will usually find him out in the end.

### THE EFFECT OF TRANSLUMINAL ANGIOPLASTY ON THE NEED FOR SURGERY IN AORTO-ILIAC OCCLUSIVE DISEASE

**Alun Davies, P. Ramarakha, Jack Collin, Peter J. Morris**
**John Radcliffe Hospital, Oxford**

Percutaneous transluminal angioplasty (PTA) would be expected to decrease the need for surgery in aorto-iliac occlusive disease.

Over the four years 1985 to 1988, 192 patients (149 men, 43 women) of median age 66 years (range 43–91) were treated for aorto-iliac occlusive disease. Eighty-one (42%) were treated by PTA alone and 111 by operation.

In 1985 only 2 patients were treated by PTA. In 1987–1988 47% of patients were treated by PTA but the number of operations increased slightly to 33 per annum as the total workload doubled. During the whole period a constant proportion of patients were treated by extra-anatomic bypass. The introduction of PTA reduced the proportion of patients treated by direct aorto-iliac surgery which in 1985 comprised 61% of all treatments but only 25% in 1986–88.

PTA has generated its own workload and although the proportion of patients treated operatively has fallen substantially the total number of operations has increased due to a stimulation of referrals combined with a relaxation of criteria for angiography.

### ILIO-FEMORAL CROSS-OVER GRAFTS

**D.A. Shields, M.C. Mason, C.P. Gibbons**
**Morriston and Singleton Hospitals, Swansea**

Increasing use of angioplasty led us to review our indications for cross-over grafting, and to substitute ilio-femoral for femoro-femoral grafting (8 of 10 cross-over grafts in the past year), as suggested by Baker and Barrie (Br. J. Surg. 1989, Vol. 76, March, 307). A muscle splitting retroperitoneal approach is employed, and good access can be obtained with a ring retractor. The graft is tunnelled behind the rectus using the superior pubic rami and symphysis pubis as a guide. We have routinely used knitted 8 or 10mm Dacron grafts, anticoagulated patients intra-operatively and used aspirin and dipyridamole subsequently. None of the grafts have needed revising, and of the six patients still alive all grafts are currently patent. In our small series there is no increase in morbidity or mortality with this method, equally good long-term patency rates should be achieved, and it allows for subsequent easy access to the donor femoral artery if required.

### THE NON-INVASIVE DETECTION OF 'AT RISK' FEMORO-DISTAL GRAFTS

**M.G. Wyatt, M. Horrocks**
**Vascular Studies Unit, Bristol Royal Infirmary**

Patency rates of femoro-distal grafts can be improved if stenoses are identified and corrected prior to graft failure. Graft surveillance using ankle pressures is disappointing, with approximately half of lesions greater than 50% missed. Duplex scanning can detect body of graft lesions, but gives less information concerning distal graft and run-off stenoses.

Impedance analysis is a new technique which can detect both graft and run-off stenoses. In 115 grafts, it successfully detected 46 of 49 stenoses greater than 50%, as shown in intra-arterial digital subtraction arteriography. It is non-invasive, inexpensive and easy to apply. The results are repeatable and impedance analysis is able to detect those 'at risk' grafts requiring further arteriographic investigation.

### PULSES, PRESSURES AND PEOPLE

**T.R. Magee, P.R.W. Stanley, R.A. Al Mufti, L. Simpson, W.B. Campbell**
Royal Devon and Exeter Hospital, Exeter

Observer variation in the palpation of pulses was investigated, comparing and contrasting this with Doppler assessment.

Seventy six limbs (33 claudicant patients and 5 controls) were examined by four observers. Dorsalis pedis and posterior tibial pulses were palpated and then examined by Doppler.

There was good agreement in assessment of normal subjects.

In claudicants there was better agreement in palpation of DP pulse (all agreed in 67%), than PT pulse (all agreed in 53%). By contrast there was better agreement on Doppler signals from PT artery (all agreed in 78%) compared with DP artery (all agreed in 58%).

In measuring pressures of claudicants variation was within ± 0.15 in 88% of limbs.

Palpation of foot pulses is subject to substantial observer error and Doppler examination is preferable at all times.

### IN-PATIENT DATA COLLECTION FOR THE GENERAL/VASCULAR SURGEON

**D. Wilkins**
**Derriford Hospital, Plymouth**

There is increasing pressure being placed upon surgeons to collect regular, accurate and timely data concerning their clinical activity. Several computer systems have been developed and marketed to fill this need but the organisation of data collection is at least as important as the technical equipment. A system that interfaces with the hospital network has been developed in Plymouth and was presented together with a brief discussion on the key aspects of setting up such a system.

